# The York County Hospital

**Published:** 1913-03-29

**Authors:** Mabel Cicely Wells

**Affiliations:** 39 St. Stephen's Mansions, Westminster, S.W.


### The Editor's Letter-Box.

### The York County Hospital.

*To the Editor of* The Hospital.

Sir,—I should be very grateful if you would insert the following in The Hospital this week. The serious, and in some cases untrue, charges brought against the York County Hospital by the late resident doctors can only be contradicted and explained by those who have worked in and for the welfare of this hospital. I have just finished my three years' training at the York County Hospital. During that time I have been thoroughly well taught and always received the utmost kindness from matron. Three times I had slight ailments, but I was never off duty, but on each of these occasions I saw the honorary doctors; the nurses have always been and are well cared for when ill, and there is no hesitation on their part to go to their ward sister or to matron when necessary. The food is excellent, nicely served, and the nurses' appetites are extraordinarily good.

Resident doctors should uphold and encourage an etiquette and atmosphere congenial to the working of the hospital and the welfare of the patients; in several incidents personally known to me Mr. Macqueen and Mr. Shepherd by no means did this. I should like to add that my three years at the York County Hospital has been extremely happy.

I write this quite independently and in answer to the slur cast upon the treatment of the nurses, and very much hope this letter will be made public.—Yours truly,

(Signed) Mabel Cicely Wells.

39 St. Stephen's Mansions, Westminster, S.W.,
March 18, 1913.

